# Self-Confidence, Satisfaction, and Knowledge of Nursing Students with Training in Basic Life Support in Pregnant Women: A Cross-Sectional Study

**DOI:** 10.3390/nursrep13010028

**Published:** 2023-02-21

**Authors:** Rocío Adriana Peinado-Molina, Sergio Martínez-Vázquez, José Félix Paulano-Martínez, Antonio Hernández-Martínez, Juan Miguel Martínez-Galiano

**Affiliations:** 1 Department of Nursing, University of Jaen, 23071 Jaen, Spain; 2 Hospital of Jaén, 23006 Jaen, Spain; 3 Department of Nursing, Faculty of Nursing of Ciudad Real, The University of Castilla-La Mancha, 02008 Ciudad Real, Spain; 4 Consortium for Biomedical Research in Epidemiology and Public Health (CIBERESP), 28029 Madrid, Spain

**Keywords:** basic life support (BLS), nursing, training, pregnant women, flipped classroom

## Abstract

Background: A flipped classroom integrating clinical simulation has been shown to be effective for basic life support (BLS) competencies in nursing students. Cardiopulmonary arrests (CPAs) in pregnant women have a low incidence but high morbidity and mortality. Current trends show an increasing incidence; however, most official university nursing training curricula do not include specific training modules for BLS in pregnant women. This study aims to know the satisfaction and self-confidence of nursing students with respect to a training intervention regarding in BLS in pregnant women. Additionally, it aims to assess the adequacy of this intervention for acquiring the necessary knowledge on the subject. Methods: A cross-sectional study was conducted at the University of Jaen in 2022. Data were collected on sociodemographic factors, previous contact with the topic, and topic knowledge in addition to the use of an SCLS questionnaire to measure satisfaction. Participants took the BLS training (a flipped classroom integrating clinical simulation on this topic) before answering the questionnaire. Results: A total of 136 students participated. The mean score on the BLS questionnaire was 9.10 out of 10 (SD = 1.01). The mean score for the SCLS questionnaire for females was 62.36 (SD = 7.70) and 56.23 (SD = 16.94) for the male group. Age showed a statistically significant association with SCLS score: the score decreased with an increase in age (*p* < 0.001). Conclusions: The flipped classroom, integrating simulation for BLS in pregnant women, improves self-confidence, satisfaction, and knowledge on the topic.

## 1. Introduction

Clinical simulation in the training of nursing students is a widely used and developed educational strategy [1,2]. This tool does not replace standard clinical practice but rather complements, innovates, and enriches the curriculum of health science disciplines. Furthermore, it provides new teaching and learning opportunities, increases patient safety, and improves students’ confidence during university education [3]. The European Higher Education Area (EHEA) recommends the use of new educational methodologies in addition to the evaluation of student satisfaction [4,5]. According to different studies [2,6], nursing students view simulation as an important learning method, and this increases their satisfaction, improves self-confidence, and contributes to reducing the gap between theoretical training and clinical practice.

The use of new pedagogical techniques is taking place in higher education. One of these new techniques is the “flipped classroom”, which has already demonstrated results [7,8,9,10]. It is essential to understand the needs of the student body [11,12]. Thus, different tools, including the Student Satisfaction and Self-Confidence in Learning Scale (SCLS), have been developed to measure student satisfaction and self-confidence through high-fidelity simulations [13]. Furthermore, these tools provide information about which factors are associated with a low level of self-confidence in students and, therefore, how they may be acted upon, improving learning results and improving the students’ learning experience in the clinical environment.

Cardiopulmonary arrests (CPA) in pregnant women have a low incidence but high morbidity and mortality. Therefore, there is a need to train future health workers in basic life support (BLS) in pregnant women. In 2003, the incidence of maternal CPA was 1/30,000 pregnancies. Current data show that 1/12,000 hospitalizations in the US are complicated by maternal CPA [14]. This increment can be explained by the increase in the mean age of pregnant women, a higher incidence of chronic diseases and cardiovascular risk factors, maternal obesity, and the increased survival of women with congenital heart disease who reach gestational age [14]. Other authors report higher incidence figures, such as Beckett et al., with an incidence of 2.78 per 100,000 pregnancies in their study carried out in 2017 [15]. There is a clear upward trend in the figures in recent years. However, most official university nursing training curricula do not include specific training modules for BLS in pregnant women [16]. It is necessary to emphasize the need for early and correct care to increase maternal survival, including the adaptations and changes that occur concerning BLS in an adult patient, and knowing these differences and correctly performing the technique is essential [17,18]. There is sufficient evidence on the importance of training using clinical simulation, as well as the importance of training future health professionals in BLS in pregnant women [19]. Thus, this study aimed to determine the satisfaction and self-confidence of nursing students regarding a training intervention regarding BLS in pregnant women and to assess the adequacy of this intervention to acquire the necessary knowledge on the subject.

## 2. Methodology

A cross-sectional descriptive study was carried out at the University of Jaen during the 2022–2023 academic year.

### 2.1. Design and Selection of Subjects

The sample consisted of second-year Nursing Degree students, selected in a non-random sampling method, who were enrolled in the second academic year in 2022 prior to receiving BLS in pregnant women training.

### 2.2. Information Sources

A self-administered questionnaire was used. The questionnaire included an informed consent form that all the students had to read and later accept before continuing with its completion. The educational intervention on BLS in pregnant women was carried out using the “flipped classroom” methodology [20]. The “flipped classroom” is a pedagogical approach that transforms the group space into a dynamic and interactive learning environment in which the educator guides the students to apply the concepts and participate creatively in the training session [7,8]. This methodology has proven to be more effective than conventional methods for teaching nursing techniques and skills [9,10,21]. The pedagogic aims of the clinical simulation were to match the benefits of a “flipped classroom”; thus, a combination of those teaching techniques was selected [7,8,9,10].

Subsequently, students were provided with a questionnaire that was structured in three parts. The first part collected data on the sociodemographic variables of the sample, such as age, sex, highest academic year in which they are currently enrolled, previous experience in the clinical environment, and whether there were pending matters from previous courses. The “Student Satisfaction and Self-confidence in Learning (SCLS) validated in Spanish” scale, which consisted of 13 Likert-type items scored from 1–5 (1: Disagree—5: Agree) was also included. Thus, this tool was validated and reliable in the population of the study sample. The SCLS measures the level of satisfaction and self-confidence of nursing students in clinical simulation. The score is directly related to self-confidence without a cut-off point, indicating that higher scores are accountable for a higher self-confidence of the students [13]. Finally, a 10-item questionnaire dedicated to assessing the degree of acquisition of knowledge obtained by the students in the field of BLS in pregnant women was included, using the latest indications on BLS from the European Resuscitation Council (ERC) 2021 [16]. This questionnaire matched the minimum requirements for passing ERC training in BLS. To pass the questionnaire, at least 5 out of 10 questions had to be correct [16].

### 2.3. Statistical Analysis

First, descriptive statistics were performed with absolute and relative frequencies for the categorical variables and the mean with the standard deviation for the quantitative variables. This type of analysis was also performed for the responses to the BLS questionnaire. Next, a bivariate analysis was carried out to study the relationship between the sociodemographic and academic profile variables with the BLS and SCLS scores, using the Student–Fisher t-tests for the dichotomous variables and an analysis of variance for those that presented more than two categories. The Pearson correlation coefficient was used for the quantitative variables. All statistical analyses will be performed with SPSS.

### 2.4. Ethical Considerations

All participants signed an informed consent prior to take part in the study. A favorable opinion was obtained from the Research Ethics Committee of the University of Jaen with reference DIC.22/3. PRY.

## 3. Results

A total of 136 students participated. The mean age was 22.34 (SD = 6.70), and the participants went through the flipped classroom training before completing the questionnaire. Most students were female, comprising 83.8% (114) of the class. Of the students, 36.8% (50) acceded university through professional education, and 58.1% (79) took the university access exam. Of the sample, 80.9% (110) had passed all their subjects. Nutrition and food was the least-passed subject, with six of the students not passing the class. Most students, 70.6% (90), had no previous clinical experience, and 94.4% (129) of the students had no BLS training. Only 8.1% (11) of the sample had previously seen BLS attempted. In addition, 91.9% (125) of the students had never attempted either a simulation or a real case of BLS. Other sociodemographic data can be seen in Table 1. 

Concerning BLS knowledge, the mean score was 9.10 out of 10 (SD = 1.01). The most failed questions after training were those related to the order of actions, with a total of 36.8% (50) of the students not answering correctly. The algorithm to follow was next, with 11% (15) of students giving incorrect answers, and the compressions technique saw 13.2% (18) provide incorrect answers. Conversely, the questions receiving the greatest number of correct answers were based on assessing a BLS situation, with 97.8% (133) correct answers provided. The use of semiautomatic defibrillation and the location of electrodes achieved the same results, and the compressions/ventilation rate question received 100% (136) correct answers. The rest of the information can be seen in Table 2.

**Figure 1:**
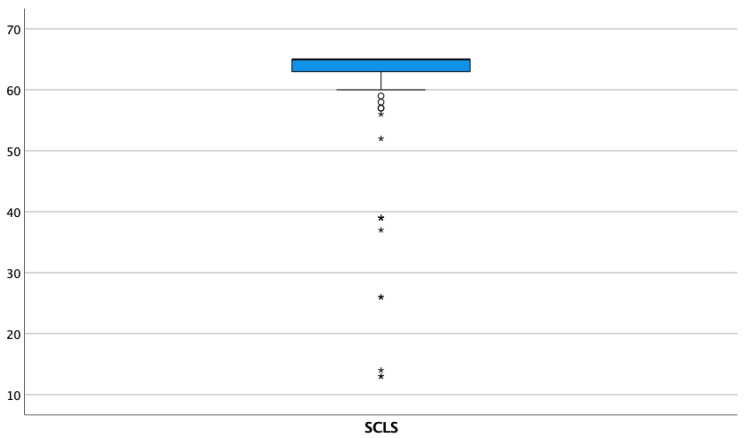
SCLS score boxplot. The asterisks mean abnormally far values (distance on the median greater than 3 interquartile ranges).

The mean score on the SCLS questionnaire was 61.38 (SD = 10.02) (Figure 1), showing higher scores in the absence of previous clinical experience with a mean of 62.73 (SD = 7.56) for the group with no experience and 58.13 (SD = 13.77) for those with previous contact with the clinical environment. In addition, those who had previously witnessed an attempt at BLS had a mean of 61.73 (SD = 9.19), and those who had not witnessed an attempt at BLS had a mean of 57.36 (SD = 16.59). The mean score for female students was 62.36 (SD = 7.70); for the male students, it was 56.23 (SD = 16.94). The age showed a statistically significant association with the SCLS score, with a decrease in scores associated to an increase in age (*p* < 0.001).

**Figure 2:**
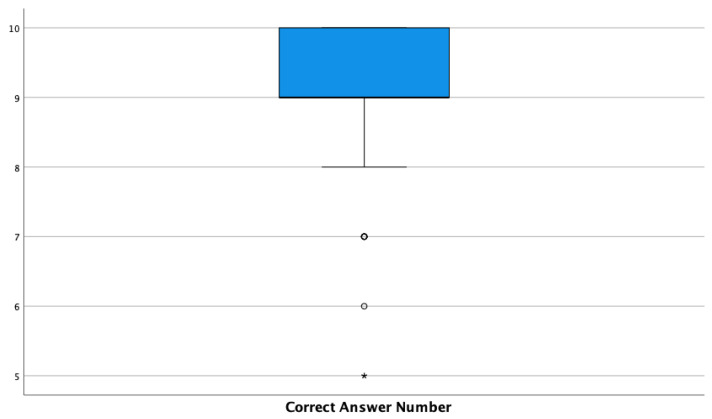
Knowledge regarding BLS in pregnant women: questionnaire score boxplot. The asterisks mean abnormally far values (distance on the median greater than 3 interquartile ranges).

The mean score of the BLS questionnaire was 9.10 (SD = 1.01) (Figure 2). Despite not showing any statistically significant association with any of the items of the questionnaire, a difference can be appreciated between the group who had previously attempted BLS, with a mean of 9.15 (SD = 0.99), and those who had not previously attempted BLS, with a mean of 8.54 (SD = 1.04). The same trend can be noted regarding previous BLS training, with a mean of 9.12 (SD = 1.03) for the students with previous training and a mean of 8.86 (SD = 0.38) for those without. It also seems remarkable that previous clinical experience positively influenced the scoring means of the BLS knowledge questionnaire. Thus, although differences existed, no statistical meaning was found. The other results can be seen in Table 3.

## 4. Discussion

After the BLS training, the average score on the BLS knowledge test was higher than 9 out of 10, demonstrating the effectiveness of the flipped classroom regarding training for BLS for pregnant women as an educational intervention that increased the degree of knowledge and that led to the achievement of objectives or learning outcomes. Concerning the SCLS questionnaire, the mean score was 61.38 after the BLS training. The score on this questionnaire was associated with having had previous experience in a clinical setting or having witnessed a case of BLS, both of which increased the mean score on the SCLS. The age showed a statistically significant association with SCLS score, with scores decreasing when age increased.

The study sample is representative of the reference population, as 94.4% of all enrolled students participated. In addition, the mean age and gender of the participants coincided with those of Spanish university students [22]. The data collection was carried out anonymously in a non-random sampling method (with its limitations), using an online questionnaire. This allowed students to respond more honestly, thus avoiding possible biases that could be produced by giving conditioned answers [23,24]. As a possible limitation of the study, the results of the SCLS may be influenced by the time of the assessment (straight after training finished). However, the scale is validated and was used in similar contexts and populations with similar measuring points [13]. We consider its effect to be minimal.

The results of the study reported that the flipped classroom was adequate for acquiring knowledge about BLS in pregnant women; however, we cannot compare the results with other studies because similar investigations that address the subject have not been identified. Nonetheless, other authors found that simulation and training in an application similar to the one carried out in our study (that is, a flipped classroom) improves knowledge about BLS (in non-pregnant adult patients) in nursing students [25,26]. Conversely, Hernández Padilla et al. [27], stated that for this training to be effective and generate skills and competencies in BLS, at least three months must elapse with these competencies remaining valid for it to be considered a training success. Our evaluation was at the end of the training session because the students would have already begun clinical practice in health centers if the evaluation was performed after three months, making it more difficult to access them. Additionally, the results could be influenced by the experience acquired in clinical training and the theory that they would have received during that period.

The mean score on the SCLS scale was higher than the scores obtained by other authors in similar contexts [28,29]. The fact that higher average scores were found may be due to the application of the flipped classroom training or a teaching model that enhances these aspects in the student learning process [9,10,20,21]. Keeping this in mind, it seems that the simulation, practice, and training of skills for practical cases improved student satisfaction, self-assessment, and self-confidence [29,30,31].

The results obtained show an increased scoring for those who had previous experience in the clinical environment and had not witnessed a case (simulated or real) of BLS. BLS is frequently addressed and taught in first-aid courses aimed at the general population. Although these results may be somewhat contradictory, they align with other authors [32,33]. The age showed a statistically significant association with SCLS score, with an increase in age associated with decreased scoring (*p* < 0.001). However, we could not compare these results with the results of other authors. Although Albagawi et al. [34] found an association between the year level and the scoring in the SCLS questionnaire, this may not be affected by the age of the nursing student other than the association between more courses being finished and an older age.

There was a tendency to obtain higher scores on the BLS knowledge questionnaire among nursing students who had prior training or had witnessed or attempted CPR in a simulated or real case. Having only witnessed the technique appeared to increase satisfaction with learning, which is in line with what was found by other authors [35,36,37]. Vicarious learning proved to be effective in the training of nursing students [36]. Other authors also included it as a key element for subsequent simulation, as it is an element that enhances learning [35,37]. As a future research line, it would be interesting to study the training effect in other nursing degree students from other contexts or even from other courses to understand the impact on self-confidence and, ultimately, the knowledge acquired.

## 5. Conclusions

Most of the students acquired BLS knowledge and were satisfied with the experience. An association was found between the level of satisfaction and self-confidence in learning during clinical simulation and training and having previous experience in a clinical environment or having witnessed a BLS case. Knowing that this approach is effective to improve the knowledge, satisfaction, and self-confidence in nursing students when learning about BLS, it could be applicable to other courses and even with qualified nurses. This would refresh techniques or aid in the learning of new skills, which may not be undertaken often, affecting the confidence of nursing professionals.

## Figures and Tables

**Table 1:** Sociodemographic characteristics of the sample.

Variable	Total
	N (%)
Age	
Mean (SD)	22.34 (6.70)
Sex	
Male	22 (16.2)
Female	114 (83.8)
Academic level	
High School	1 (0.7)
Professional Education	50 (36.8)
University Access	79 (58.1)
Degree	5 (3.7)
Master’s degree	1 (0.7)
All subjects passed	
No	26 (19.1)
Yes	110 (80.9)
Subjects not passed (more frequent)	
Nutrition and Food	6 (4.4)
Psychosocial and Human Relations	4 (2.9)
Biostatistics	5 (3.7)
Both Psychosocial and Human Relations and Biostatistics	2 (1.5)
Previous experience in a clinical environment	
No	96 (70.6)
Yes	40 (29.4)
Previous BLS training	
No	129 (94.4)
Yes	7 (5.1)
Previously seen BLS	
No	125 (91.9)
Yes	11 (8.1)
Previously attempted BLS	
No	125 (91.9)
Yes	11 (8.1)

**Table 2:** BLS in pregnant women questionnaire after training.

Questionnaire of Knowledge (after Training)	Total
	N (%)
What is BLS?	
Incorrect	7 (5.1)
Correct	129 (94.9)
The correct algorithm in BLS	
Incorrect	15 (11.0)
Correct	121 (89.0)
Order of actions in BLS	
Incorrect	50 (36.8)
Correct	86 (63.2)
Assessing a BLS situation	
Incorrect	3 (2.2)
Correct	133 (97.8)
Use of semiautomatic defibrillation (moment)	
Incorrect	12 (8.8)
Correct	124 (91.2)
Use of semiautomatic defibrillation (electrodes location)	
Incorrect	3 (2.2)
Correct	133 (97.8)
Ventilation/compressions rate	
Incorrect	0 (0.0)
Correct	136 (100.0)
Compressions technique	
Incorrect	18 (13.2)
Correct	118 (86.8)
Initial pathway of BLS (when conscious and breathing)	
Incorrect	5 (3.7)
Correct	131 (96.3)
Initial pathway of BLS (when unconscious and breathing)	
Incorrect	9 (6.6)
Correct	127 (94.3)

**Table 3:** Relationship between sociodemographic and academic variables with SCLS and BLS Knowledge.

Variable	SCLS Mean (SD)	*p*-Value	BSL Mean (SD)	*p*-Value
** Age**	−0.278	**0.001** ^a^	0.005	0.957 ^a^
** Sex**		0.109 ^b^		0.874 ^b^
Female	62.36 (7.70)		9.09 (1.00)	
Male	56.23 (16.94)		9.14 (1.08)	
**Academic level**		0.526 ^c^		0.987 ^c^
High School	62.08 (8.71)		9.06 (0.94)	
Training (Module)	61.10 (10.16)		9.16 (1.50)	
Secondary School	57.00 (NC)		9.00 (NC)	
Previous Degree	54.20 (23.05)		9.20 (0.84)	
Postgrad	60.00 (NC)		9.00 (NC)	
**All the subjects passed**		0.935 ^b^		0.884 ^b^
No	61.23 (11.14)		9.08 (1.06)	
Yes	61.41 (9.73)		9.11 (1.00)	
**Previous experience in clinical environment**				
No	62.73 (7.56)	0.052 ^b^	9.06 (0.97)	0.492 ^b^
Yes	58.13 (13.77)		9.20 (1.09)	
**Previous BLS training**		0.981 ^b^		0.509 ^b^
No	61.38 (10.02)		9.12 (1.03)	
Yes	61.29 (9.83)		8.86 (0.38)	
**Previously seen BLS**		0.408 ^b^		0.725 ^b^
No	61.73 (9.19)		9.11 (1.02)	
Yes	57.36 (16.59)		9.00 (0.89)	
**Previously attempted BLS**		0.668 ^b^		0.055 ^b^
No	61.48 (9.40)		9.15 (0.99)	
Yes	60.09 (15.62)		8.54 (1.04)	

Bold: Statistically significant differences; a: Pearson’s correlation coefficient; b: Student-Fisher *t* test; c: Analysis of variance. NC: not calculable.

## Data Availability

All the data are available from the authors through reasonable request.
